# The 2021 Core Content of Medical Toxicology

**DOI:** 10.1007/s13181-021-00844-5

**Published:** 2021-06-29

**Authors:** Robert G. Hendrickson, Theodore C. Bania, Carl R. Baum, Michael I. Greenberg, Kevin B. Joldersma, Julia N. Keehbauch

**Affiliations:** 1 Oregon Poison Center, Fellowship in Medical Toxicology, Department of Emergency Medicine, Oregon Health and Science University (OHSU), 3181 SW Sam Jackson Park Road, CSB 550, Portland, OR 97239 USA; 2 Department of Emergency Medicine, Mt Sinai West, Icahn School of Medicine at Mount Sinai, 1000 10th Ave. Rm GE01, New York, NY 10019 USA; 3 Pediatrics and Emergency Medicine, Yale School of Medicine, 100 York St, Suite 1F, New Haven, CT 06511 USA; 4 Emergency Medicine, Division of Medical Toxicology, Department of Emergency Medicine, Drexel University College of Medicine, Philadelphia, PA USA; 5 American Board of Emergency Medicine, 3000 Coolidge Road, East Lansing, MI 48823 USA

**Keywords:** Core Content, Medical Toxicology, Toxicology, Curriculum

## Abstract

The Medical Toxicology Subboard approved modifications to the Core Content of Medical Toxicology in March 2021. The document outlines the areas of knowledge considered essential for the practice of medical toxicology. The Core Content provides the organizational framework for the development of the Medical Toxicology Certification and Cognitive Expertise Examinations and serves as a template for the development of curricula for medical toxicology fellowship training programs.

## Introduction

Contemporary medical curricula strive to “achieve a symbiosis with the health services and communities in which the students will serve” [1]. A “Core Content” document defines the essential body of knowledge for a given academic discipline and forms the basis for curriculum design, education, and testing.

In 1994, the Medical Toxicology Subboard (representing the American Board of Emergency Medicine, the American Board of Pediatrics, and the American Board of Preventive Medicine) developed the first Core Content of Medical Toxicology to inform the construction of the first certifying examination. This document consisted of 22 major content areas and was organized, in part, by toxicant classification. In 2000, and again in 2009, the Medical Toxicology Subboard convened a task force charged with updating the existing Core Content document, and to improve the framework that conceptualized the expanding body of knowledge of medical toxicology. These task forces solicited input from program directors for medical toxicology fellowship training programs and medical toxicology diplomates and established an ABEM contact and e-mail for recommendations about the Core Content from all medical toxicologists [2, 3]. The resulting version of the Core Content was published in 2012.

In 2020, the Medical Toxicology Subboard convened a task force charged with evaluating, refining, and updating the 2012 Core Content of Medical Toxicology. The 2020 Medical Toxicology Core Content Task Force was comprised of four board-certified medical toxicologists with specific content expertise in medical toxicology, pediatrics, environmental toxicology, emergency medicine, occupational toxicology, preventive medicine, poison center administration, emergency/disaster management, addiction medicine, and medical education. The task force members included current and former program directors from the US medical toxicology fellowship training programs, as well as staff members from the American Board of Emergency Medicine with expertise in psychometrics and examination design and development.

The 2020 task force considered the future needs of the specialty and recognized that a shortage of medical toxicologists currently exists. Many US academic and non-academic centers alike do not have medical toxicologists performing inpatient or outpatient consultations. As of 2020, the number of medical toxicology fellows enrolling in fellowship training programs is increasing; however, 25–33% of total positions in the country remain unfilled, and 33–50% of programs are unfilled each year [4]. The 2020 task force acknowledged that one way to encourage young physicians to consider careers in medical toxicology is to ensure that the Core Content remains current and relevant with regard to the current practice of medical toxicology.

In February 2021, the Medical Toxicology Subboard approved a revised Core Content document. The 2021 Core Content of Medical Toxicology encompasses the body of knowledge for the specialty of medical toxicology and outlines the areas of knowledge considered essential for its practice. The 2021 Core Content provides the organizational framework for the development of the Medical Toxicology Certification and Cognitive Expertise Examinations and details the knowledge to be tested beginning with the 2022 examination. In addition, the Core Content serves as a template for the development and application of curricula for fellowship training programs in medical toxicology. The current revision replaces the 2012 Core Content of Medical Toxicology.

## Methodology

The methodology applied in the development of the 2021 Core Content involved detailed analysis of the previous Core Content documents and ultimate consensus among the task force members. The work of the 2020 task force was informed by the knowledge that the Core Content is used by learners to direct their study, by program directors to direct their teaching, by test item writers to determine question topics, and by test designers to assemble the Certification and Cognitive Expertise Examinations.

The American Board of Emergency Medicine surveyed, via e-mail, 201 board-certified medical toxicologists with a wide variety of years-of-practice, practice setting, academic affiliation, medical publishing/research, involvement in teaching fellows, and poison center affiliation. Medical toxicologists rated how often each aspect of the 2012 Core Content was encountered and how important each item was to their current practice of medical toxicology. Survey respondents were also able to submit additional comments to inform this process. The survey was analyzed by the task force to identify areas for expanded or restricted detail. The task force expended in excess of 250 person-hours in the analysis and re-drafting of the Core Content.

The 2020 task force analyzed the 2012 Core Content document, which was organized into six subject areas: (1) principles of toxicology, (2) toxins and toxicants, (3) clinical assessment, (4) therapeutics, (5) assessment and population health, and (6) analytical and forensic toxicology. The 2020 task force expanded the “top-level” subject areas to include (7) environmental toxicology, (8) occupational and industrial toxicology, and (9) addiction toxicology and substance use. The intent was to improve the organization of the Core Content and to acknowledge the importance of these domains within the discipline of medical toxicology. The task force further sought to create a document that reflects current medical toxicology practice.

The Core Content was divided into sections for initial review by two task force members to recommend potential edits based on the guiding principles. Each recommendation was discussed by all four task force members, and the determination of consensus among task force members was used to finalize edits. Task force members then reviewed the entire document for consistency and presented the draft Core Content to the entire Medical Toxicology Subboard. After final editing and cross-referencing, the final Core Content document was approved by the Medical Toxicology Subboard.

## Discussion

The 2020 task force defined a set of “guiding principles” to be used in updating the Core Content, which included decreasing ambiguity and increasing the clarity and clinical relevance of the Core Content document.

### Decreasing Ambiguity

The 2020 task force recognized that the Core Content may be used to design educational fellowship programs and to provide a guide for learners to organize their education and study. For this reason, the task force aimed to decrease ambiguities in the document. The task force drastically reduced the use of “examples” throughout the document to eliminate the implication that these “examples” were more important than other line items in a category. For example, the subsection that formerly listed “Specific radioisotopes (e.g., cesium, iodine, polonium, radon)” was updated to remove the “e.g.,” and now specifically lists the radioisotopes that a medical toxicologist should be familiar with and understand. This removes the implication that cesium, iodine, polonium, and radon are more important than others but also limits the number of clinically relevant radioisotopes that a medical toxicologist must master. Similarly, the task force decreased the use of the term “others” in the document, as this may imply that medical toxicologists must know all possible aspects of a topic and that any aspect of that topic may be a potential question on an examination. For example, in the section “metals/metalloids,” the metals and metalloids that are necessary to understand are specifically listed in place of “other.” As practice evolves, additional items may be added in subsequent revisions to the document. The Core Content is a guideline and, while no guideline encompasses every aspect of a topic, the task force felt that it was important to be specific when possible.

### Increased Clarity

Revisions to some sections were made to specify the knowledge that a medical toxicologist should have about expansive topics. For example, “Role of the Medical Review Officer” was eliminated in favor of including additional topics under “interpretation of drug testing” that encompass aspects of the medical review officer role in current medical toxicology practice. Similarly, “confirmatory test; gas chromatography” was changed to “interpretation of confirmatory test results; gas chromatography” to de-emphasize the technical aspects of the test (i.e., how the lab performs the test) and emphasize the interpretation of the test and its advantages and limitations.

### Clinical Relevance

A primary goal of the 2020 task force was to reorganize the Core Content focusing on clinical relevance. Certain sections were compressed and others were expanded to be more consistent with their relative weight in current medical toxicology practice. The task force added sections that have increasing importance in medical toxicology, including QRS prolongation, QTc prolongation, neonatal abstinence syndrome, epigenetics, and extracorporeal membrane oxygenation (ECMO). “Cannabis” was removed from the category “Psychoactive Drugs and Hallucinogens” and placed into its own category, given its prominence in the United States and the increasing legalization of cannabis.

The Occupational/Industrial Toxicology section was organized according to occupation and industry, with pertinent chemicals listed as subsections, because a medical toxicologist often evaluates workers from a particular occupation or industry (e.g., welder, painter, petrochemical industry worker) rather than with a specific chemical exposure (e.g., carbon disulfide exposure).

The task force further attempted to de-emphasize certain areas of the 2012 Core Content that were prone to esoterica. For example, the section “Regulatory and legal background (e.g., HAZWOPER, SARA, CERCLA, RCRA, etc.)” was removed; while the application of these laws may be integral to the practice of medical toxicology, the specifics of the law (wording, date of enactment, etc.) are de-emphasized. The task force removed “miscellaneous toxicants” and moved those chemicals to a new “occupational/industrial toxicology” section to more specifically contextualize them. Further, the 2020 task force agreed by consensus to eliminate from the Core Content document chemicals that have been banned or unavailable in the United States for at least 50 years and are not typically used internationally. These chemicals have been placed into the section entitled “Exposures of Historical Significance.”

The 2020 task force added three domains to the Core Content that reflect current medical toxicology practice: Environmental Toxicology, Occupational/Industrial Toxicology, and Addiction Toxicology. The intent of adding these sections was to reorganize existing sections of the Core Content and group them to more accurately reflect the current practice of the specialty of medical toxicology. Much of the content for these sections was contained in the 2012 Core Content document, but is re-organized to maximize clinical relevance.

The 2021 Core Content is intended to be a “living document,” in keeping with the evolving practice of medical toxicology. The Subboard anticipates periodic updates to the Core Content and recommends that these updates occur approximately every 5 years.

## The 2021 Core Content of Medical Toxicology

### Part 1: PRINCIPLES OF TOXICOLOGY

1.1 Principles of Pharmacology/Toxicology

1.1.1 Pharmacokinetics/Toxicokinetics

   1.1.1.1 Bioavailability and absorption

   1.1.1.2 Clearance

   1.1.1.3 Distribution

   1.1.1.4 Elimination

   1.1.1.5 Metabolism

   1.1.1.6 Pharmacokinetic modeling

1.1.2 Pharmacodynamics/Toxicodynamics

   1.1.2.1 Dose/Concentration relationship to effect

   1.1.2.2 Receptor agonism/Antagonism

   1.1.2.3 Receptor regulation

   1.1.2.4 Structure-activity relationship

1.1.3 Adverse drug effect

1.1.4 Interactions

   1.1.4.1 Drug-drug

   1.1.4.2 Xenobiotic

1.1.5 Immunologic response

1.1.6 Pharmacogenetics/Toxicogenetics

   1.1.6.1 Polymorphisms causing adverse effects

1.2 Principles of Molecular Components/Mechanisms

1.2.1 Ion channels and ion pumps

1.2.2 Enzymes and transport proteins

1.2.3 Glycolysis and oxidative phosphorylation

1.2.4 Membranes

1.2.5 Neurotransmitters (see 8.4)

1.3 Principles of Radiation

1.4 Principles of Mutagenesis and Carcinogenesis

1.4.1 Mutagenesis

1.4.2 Carcinogenesis

   1.4.2.1 Procarcinogens

   1.4.2.2 Initiation

   1.4.2.3 Progression

   1.4.2.4 Promotion

1.5 Principles of Epigenetics

1.6 Principles of Reproductive and Developmental Toxicology

1.6.1 Conception impairment/Mutagenesis/Teratogenesis

1.6.2 Pharmacology of pregnancy

1.6.3 Factors determining fetal or infant exposure to agents

   1.6.3.1 Placental transfer

   1.6.3.2 Fetal pharmacokinetics

   1.6.3.3 Breast milk transfer

1.6.4 Pharmacology of the neonate

### Part 2: TOXICANTS AND TOXINS

2.1 Medications and Drugs

2.1.1 Analgesics/Anti-inflammatory drugs

   2.1.1.1 Acetaminophen

   2.1.1.2 NSAIDs

   2.1.1.3 Opioids (see 3.1.3)

   2.1.1.4 Salicylates

   2.1.1.5 Colchicine

2.1.2 Antimicrobials

   2.1.2.1 Antibiotics (see 7.2.3.3.2)

   2.1.2.2 Antifungals

   2.1.2.3 Antimycobacterials

   2.1.2.4 Antiparasitics

   2.1.2.5 Antiprotozoals

   2.1.2.6 Antiretrovirals

   2.1.2.7 Antiseptics

   2.1.2.8 Antivirals

2.1.3 Chemotherapeutic drugs

   2.1.3.1 Alkylators

   2.1.3.2 Antimetabolites

   2.1.3.3 Hormones

   2.1.3.4 Antimitotics

   2.1.3.5 Monoclonal antibodies

   2.1.3.6 Platinum-based

   2.1.3.7 Protein kinase inhibitors

   2.1.3.8 Topoisomerase inhibitors

2.1.4 Diagnostic contrast agents

2.1.5 Drugs affecting lipids

2.1.6 Drugs affecting the cardiovascular system

   2.1.6.1 Cardiovascular

     2.1.6.1.1 Calcium channel blockers

     2.1.6.1.2 Cardioactive steroids

     2.1.6.1.3 Potassium channel blockers

     2.1.6.1.4 Sodium channel blockers

   2.1.6.2 Antihypertensives

     2.1.6.2.1 Angiotensin system modulators

     2.1.6.2.2 Peripheral alpha antagonists

     2.1.6.2.3 Beta adrenergic antagonists

     2.1.6.2.4 Centrally acting alpha receptor agonists

     2.1.6.2.5 Diuretics

     2.1.6.2.6 Vasodilators

2.1.7 Drugs affecting the respiratory system

   2.1.7.1 Bronchodilators and respiratory stimulants

   2.1.7.2 Antitussives and cold preparations

2.1.8 Drugs affecting the endocrine system

   2.1.8.1 Antidiabetic drugs

   2.1.8.2 Drugs affecting bone metabolism

   2.1.8.3 Electrolytes and minerals

   2.1.8.4 Glucocorticoids

  2.1.8.5 Sex hormones/Growth hormones/Anabolic steroids

   2.1.8.6 Thyroid drugs

   2.1.8.7 Vasopressin and somatostatin analogues

2.1.9 Drugs affecting the gastrointestinal system

   2.1.9.1 Antidiarrheals

   2.1.9.2 Antiemetics

   2.1.9.3 Drugs used for biliary and pancreatic diseases

   2.1.9.4 Drugs used for inflammatory bowel disease

   2.1.9.5 Drugs used to treat esophageal-peptic disorders

   2.1.9.6 Laxatives

   2.1.9.7 Promotilics

2.1.10 Drugs that affect the hematologic system

   2.1.10.1 Anticoagulants

   2.1.10.2 Antifibrinolytics

   2.1.10.3 Antiplatelet drugs

   2.1.10.4 Bone marrow stimulating drugs

   2.1.10.5 Drugs used to treat bleeding

   2.1.10.6 Iron

   2.1.10.7 Thrombolytics

2.1.11 Drugs that affect the immune system

   2.1.11.1 Corticosteroids

   2.1.11.2 Immunosuppressants

   2.1.11.3 Biologics

2.1.12 Drugs that affect the nervous system

   2.1.12.1 Anesthetics

     2.1.12.1.1 Inhalational and sedative anesthetics

     2.1.12.1.2 Local anesthetics

   2.1.12.2 Anticonvulsant drugs

   2.1.12.3 Antiparkinsonism drugs

   2.1.12.4 Drugs that affect autonomic homeostasis

     2.1.12.4.1 Anticholinergics (see 3.1.1)

     2.1.12.4.2 Antihistamines

     2.1.12.4.3 Antiserotonergics

     2.1.12.4.4 Cholinergics (see 3.1.2)

     2.1.12.4.5 Ergot and derivatives

     2.1.12.4.6 Serotonin agonists and proserotonergics      (see 3.1.5)

   2.1.12.5 Ethanol

   2.1.12.6 Muscle relaxants

   2.1.12.7 Neuromuscular blockers

   2.1.12.8 Psychoactive drugs and hallucinogens

   2.1.12.9 Psychotropics

     2.1.12.9.1 Antidepressants

     2.1.12.9.2 Antipsychotics

     2.1.12.9.3 Anxiolytics and sedative-hypnotics

     2.1.12.9.4 Mood stabilizers

2.1.13 Pharmaceutical excipients/Diluents/Solvents

2.1.14 Veterinary products

2.1.15 Abortifacients

2.1.16 Drugs of abuse (see 8.2 and 8.8)

   2.1.16.1 CNS depressants

   2.1.16.2 Dissociatives

   2.1.16.3 Hallucinogens

   2.1.16.4 Cannabis/Cannabinoids

   2.1.16.5 Inhalants

   2.1.16.6 Opioids

   2.1.16.7 Sympathomimetics

   2.1.16.8 Diluents/Adulterants/Contaminants

   2.1.16.9 Novel psychoactive substances

2.2 Household Items

2.2.1 Cleansers and caustics

   2.2.1.1 Acids

   2.2.1.2 Alkali

   2.2.1.3 Bleach

   2.2.1.4 Detergents and soaps

   2.2.1.5 Disinfectants and topical anti-infectives

     2.2.1.5.1 Hydrogen peroxide

     2.2.1.5.2 Potassium permanganate

2.2.2 Swimming pool products

   2.2.2.1 Chlorine

   2.2.2.2 Bromine

2.2.3 Aquarium products

2.2.4 Art products

2.2.5 Batteries

2.2.6 Cosmetics

2.2.7 Dental products

2.2.8 Hair products

2.2.9 Personal hygiene products

2.2.10 Automotive products

2.2.11 Household pesticides

   2.2.11.1 DEET

   2.2.11.2 Moth repellents

   2.2.11.3 Pyrethrins and pyrethroids

2.2.12 Household rodenticides

   2.2.12.1 Hydroxycoumarins and inandiones

   2.2.12.2 Bromethalin

2.3 Supplements

   2.3.1 Herbal products

   2.3.2 Performance enhancing substances

   2.3.3 Dietary supplements

   2.3.4 Vitamins

2.4 Food Poisoning

   2.4.1 Bacterial toxins

   2.4.2 Botulism

   2.4.2.1 Infant

   2.4.2.2 Food-borne

2.4.3 Marine toxins

   2.4.3.1 Ciguatera

   2.4.3.2 Scombroid

   2.4.3.3 Tetrodotoxin

   2.4.3.4 Shellfish poisonings

     2.4.3.4.1 Amnestic

     2.4.3.4.2 Paralytic

     2.4.3.4.3 Neurotoxic

2.5 Fungi

2.5.1 Mushrooms

   2.5.1.1 Cyclopeptide

   2.5.1.2 Gyromitrin

   2.5.1.3 Muscarine

   2.5.1.4 Coprine

   2.5.1.5 Ibotenic acid and muscimol

   2.5.1.6 Psilocybin

   2.5.1.7 Gastrointestinal toxic

   2.5.1.8 Orellanine

   2.5.1.9 Allenic norleucine

   2.5.1.10 Lycoperdon

2.5.2 Fungal toxins

   2.5.2.1 Aflatoxins

   2.5.2.2 Trichothecene mycotoxins

2.6 Plants

2.6.1 Cutaneous/Mucous membrane

2.6.2 Toxalbumins

2.6.3 Sodium channel activators

2.6.4 Pyrrolizidine alkaloid

2.6.5 Nicotine-like

2.6.6 Mitotic inhibitors

2.6.7 Gastrointestinal toxins

2.6.8 Cyanogenic

2.6.9 Convulsant

2.6.10 Cardioactive steroids

2.6.11 Calcium oxalate

2.6.12 Anticholinergic

2.7 Envenomations

2.7.1 Arthropods

   2.7.1.1 Hymenoptera

   2.7.1.2 Scorpions

   2.7.1.3 Spiders

2.7.2 Marine Animals

2.7.3 Reptiles

   2.7.3.1 Venomous snakes

     2.7.3.1.1 Hemotoxic

     2.7.3.1.2 Neurotoxic

     2.7.3.1.3 Cytotoxic

   2.7.3.2 Venomous lizards

2.7.4 Amphibians

2.8 Agents of Warfare/Terrorism

2.8.1 Biological

   2.8.1.1 Bacteria

   2.8.1.2 Toxins

   2.8.1.3 Viruses

2.8.2 Chemical

   2.8.2.1 Nerve agents

   2.8.2.2 Blister agents/Vesicants

   2.8.2.3 Systemic agents/Asphyxiants

   2.8.2.4 Irritant/Corrosive

   2.8.2.5 Incapacitating agents

   2.8.2.6 Riot control agents (see 9.6.7.2)

   2.8.2.7 Opioids

2.9 Radiological

2.9.1 Radiation sources

2.9.2 Specific radionuclides

   2.9.2.1 Americium

   2.9.2.2 Cesium

   2.9.2.3 Cobalt

   2.9.2.4 Iodine

   2.9.2.5 Plutonium

   2.9.2.6 Polonium

   2.9.2.7 Radium

   2.9.2.8 Strontium

   2.9.2.9 Thorium

   2.9.2.10 Uranium

### Part 3: CLINICAL ASSESSMENT

3.1 Toxicologic Syndromes

3.1.1 Anticholinergic (see 2.1.12.4.1)

3.1.2 Cholinergic (see 2.1.12.4.4)

3.1.3 Opioid (see 2.1.1.3)

3.1.4 Sedative-hypnotic

3.1.5 Serotoninergic (see 2.1.12.4.6)

3.1.6 Sympathomimetic

3.2 Differential Diagnosis

3.2.1 Cardiovascular

   3.2.1.1 Blood pressure

   3.2.1.2 Heart rate

   3.2.1.3 Oxygenation

   3.2.1.4 Electrocardiographic

     3.2.1.4.1 Dysrhythmias

     3.2.1.4.2 QRS prolongation

     3.2.1.4.3 QT prolongation

3.2.2 Dermatologic

   3.2.2.1 Skin color abnormality

   3.2.2.2 Hair and nail abnormalities

   3.2.2.3 Rash and other cutaneous reactions

3.2.3 Gastrointestinal

   3.2.3.1 Diarrhea/Constipation

   3.2.3.2 GI hemorrhage

   3.2.3.3 Hepatic dysfunction

   3.2.3.4 Pancreatitis

   3.2.3.5 Vomiting

3.2.4 Hematologic

   3.2.4.1 Red blood cell abnormalities

   3.2.4.2 White blood cell abnormalities

   3.2.4.3 Hemostatic disorders

3.2.5 Musculoskeletal

   3.2.5.1 Rhabdomyolysis

   3.2.5.2 Skeletal abnormalities

3.2.6 Neurologic

   3.2.6.1 Ataxia

   3.2.6.2 Delirium

   3.2.6.3 Altered mental status

   3.2.6.4 Headache

   3.2.6.5 Movement disorder

   3.2.6.6 Neuropathy/Paresthesia

   3.2.6.7 Rigidity

   3.2.6.8 Seizure

   3.2.6.9 Tremor

   3.2.6.10 Weakness/Paralysis

3.2.7 Psychiatric

   3.2.7.1 Agitation

   3.2.7.2 Depression

   3.2.7.3 Hallucinations

   3.2.7.4 Psychosis

3.2.8 Pulmonary

   3.2.8.1 Cough

   3.2.8.2 Pulmonary edema/Acute lung injury

   3.2.8.3 Restrictive lung disease

   3.2.8.4 Smoke inhalation

   3.2.8.5 Wheezing/Bronchospasm

3.2.9 Renal/Genitourinary

   3.2.9.1 Kidney injury

   3.2.9.2 Nephritis/Nephrosis

   3.2.9.3 Obstruction/Renal stone

   3.2.9.4 Fertility abnormalities

   3.2.9.5 Sexual dysfunction

3.2.10 Ophthalmologic

   3.2.10.1 Vision loss/Changes

   3.2.10.2 Pupil changes

   3.2.10.3 Nystagmus

3.2.11 Otologic

   3.2.11.1 Hearing loss

   3.2.11.2 Tinnitus

3.2.12 Pediatric and Reproductive Syndromes

   3.2.12.1 Fetal abnormalities

     3.2.12.1.1 Fetal alcohol syndrome

     3.2.12.1.2 Fetal hydantoin syndrome

     3.2.12.1.3 Fetal valproate syndrome

     3.2.12.1.4 Fetal warfarin syndrome

     3.2.12.1.5 Fetal aminopterin/Methotrexate syndrome

     3.2.12.1.6 Retinoic acid embryopathy

     3.2.12.1.7 Thalidomide embryopathy

     3.2.12.1.8 Fetal solvent syndrome

   3.2.12.2 Developmental disorders

   3.2.12.3 Child abuse

3.3 Exposures of Historical Significance

### Part 4: THERAPEUTICS

4.1 Initial Management

4.1.1 Decontamination strategies

   4.1.1.1 Dermal

   4.1.1.2 Gastrointestinal

   4.1.1.3 Ocular

4.1.2 Enhanced elimination techniques

   4.1.2.1 Extracorporeal removal

   4.1.2.2 Multi-dose activated charcoal

   4.1.2.3 Urinary clearance

4.1.3 Pharmacological basis of antidote use

   4.1.3.1 Amyl and sodium nitrite

   4.1.3.2 Botulinum antitoxin

   4.1.3.3 Bromocriptine

   4.1.3.4 Calcium

   4.1.3.5 L-carnitine

   4.1.3.6 Cyproheptadine

   4.1.3.7 Dantrolene

   4.1.3.8 Deferoxamine

   4.1.3.9 Digoxin-specific fab

   4.1.3.10 Dimercaprol

   4.1.3.11 Ca- and Zn-DTPA

   4.1.3.12 Edetate calcium disodium

   4.1.3.13 Ethanol

   4.1.3.14 Flumazenil

   4.1.3.15 Folinic acid (leucovorin)

   4.1.3.16 Fomepizole

   4.1.3.17 Glucagon

   4.1.3.18 Glucarpidase

   4.1.3.19 Hydroxocobalamin

   4.1.3.20 Hyperbaric oxygen

   4.1.3.21 Intravenous lipid emulsion

   4.1.3.22 Latrodectus antivenom

   4.1.3.23 Methylene blue

   4.1.3.24 Naloxone

   4.1.3.25 N-acetylcysteine

   4.1.3.26 North American crotalidae polyvalent fab

   4.1.3.27 North American crotalidae F(ab’)_2_

   4.1.3.28 North American coral snake antivenom

   4.1.3.29 Octreotide

   4.1.3.30 Physostigmine

   4.1.3.31 Potassium iodide

   4.1.3.32 Pralidoxime

   4.1.3.33 Protamine

   4.1.3.34 Prussian blue

   4.1.3.35 Pyridoxine

   4.1.3.36 Scorpion (centruroides) antivenom F(ab’)_2_

   4.1.3.37 Sodium bicarbonate

   4.1.3.38 Sodium thiosulfate

   4.1.3.39 Succimer (2,3-dimercaptosuccinic acid)

   4.1.3.40 Thiamine

   4.1.3.41 Uridine triacetate

   4.1.3.42 Vitamin K1

4.1.4 Supportive and other care

   4.1.4.1 Airway management/Oxygenation/Ventilation

   4.1.4.2 Anticonvulsants

   4.1.4.3 Antidysrhythmics

   4.1.4.4 Control of agitation

   4.1.4.5 Control of blood pressure and heart rate

   4.1.4.6 Control of temperature

   4.1.4.7 Correction of electrolyte and acid-base disturbances

   4.1.4.8 Transplantation

   4.1.4.9 Extracorporeal membrane oxygenation

4.1.5 Radiation exposure management

   4.1.5.1 Dosimetry

   4.1.5.2 Decontamination

   4.1.5.3 Chelation therapy

### Part 5: ASSESSMENT AND POPULATION HEALTH

5.1 Causation

5.1.1 Bradford Hill associations

5.2 Principles of Epidemiology and Study Design

5.2.1 Poisoning epidemiology and study design

5.2.2 Study design

   5.2.2.1 Study types

     5.2.2.1.1 Grading of scientific evidence

     5.2.2.1.2 Non-human studies

     5.2.2.1.3 Observational studies

     5.2.2.1.4 Clinical trials

   5.2.2.2 Validity and generalizability

5.2.3 Statistical measures

   5.2.3.1 Statistical power and sample size

   5.2.3.2 Measures of association

   5.2.3.3 Test characteristics

     5.2.3.3.1 Sensitivity/Specificity

     5.2.3.3.2 Predictive values

     5.2.3.3.3 Likelihood ratios

5.3 Poison Centers

5.3.1 Administration/Organization

5.3.2 Data collection systems

5.3.3 Surveillance/Interaction with other professional health organizations

5.4 Response to Hazardous Materials (Hazmat) Incidents/Terrorism

5.4.1 Scene decontamination

5.4.2 Incident command system and control zones

5.4.3 Hazardous Materials Resources (e.g., Strategic National Stockpile, CHEMPACK program)

5.4.4 Clandestine drug laboratories

5.5 Regulatory and Legal Background

5.5.1 Role of federal and international agencies in toxicology

   5.5.1.1 Centers for Disease Control and Prevention (CDC)

   5.5.1.2 Agency for Toxic Substances and Disease Registry (ATSDR)

   5.5.1.3 National Institute for Occupational Safety and Health (NIOSH)

   5.5.1.4 Consumer Product Safety Commission (CPSC)

   5.5.1.5 Drug Enforcement Administration (DEA)

      5.5.1.5.1 Definition and scheduling of controlled substances

   5.5.1.6 Environmental Protection Agency (EPA)

   5.5.1.7 Food and Drug Administration (FDA)

      5.5.1.7.1 Risk evaluation and mitigation strategies

   5.5.1.8 Health Resources and Services Administration (HRSA)

   5.5.1.9 Occupational Safety and Health Administration (OSHA)

   5.5.1.10 World Health Organization (WHO)

       5.5.1.10.1 International Agency for Research on Cancer (IARC)

5.6 Injury Prevention

5.6.1 Poisoning prevention

5.6.2 Medication safety

   5.6.2.1 Adverse event/Adverse reaction/Medication error

   5.6.2.2 Stages of medication use

   5.6.2.3 Error reduction strategies

### Part 6: ANALYTICAL AND FORENSIC TOXICOLOGY AND WORKPLACE DRUG TESTING

6.1 Drug Testing

6.1.1 Interpretation of drug tests (see 8.6)

   6.1.1.1 Immunoassays

   6.1.1.2 Colorimetric tests

   6.1.1.3 Atomic absorption

   6.1.1.4 Gas chromatography/Mass spectrometry (GC/MS)

   6.1.1.5 High-pressure liquid chromatography (HPLC)

6.1.2 Urine drug testing

   6.1.2.1 Analytical limitations and interferences

   6.1.2.2 Interpretation of urine drug tests (see 8.6)

     6.1.2.2.1 False positives and false negatives

     6.1.2.2.2 Passive exposure to drugs of abuse

6.1.3 Additional testing matrices

   6.1.3.1 Meconium

   6.1.3.2 Vitreous humor

   6.1.3.3 Hair

6.1.4 Laboratory quality assurance/Quality control (e.g., CLIA)

6.1.5 Workplace drug and alcohol testing

   6.1.5.1 Interpretation of workplace drug testing

     6.1.5.1.1 Drug tests and impairment

     6.1.5.1.2 Cutoffs

     6.1.5.1.3 Adulterants/Diluents/Substitutions

   6.1.5.2 Principles of workplace drug testing

   6.1.5.3 Federal drug and alcohol testing rules

6.1.6 Drug testing in non-workplace settings

6.1.7 Forensic toxicology

   6.1.7.1 Postmortem specimens

     6.1.7.1.1 Selection of specimens

     6.1.7.1.2 Interpretation

     6.1.7.1.3 Redistribution

   6.1.7.2 Chain of custody

   6.1.7.3 Medical legal issues

     6.1.7.3.1 Role of expert witness

     6.1.7.3.2 Ethical standards for testimony

     6.1.7.3.3 Standards of reliability

       6.1.7.3.3.1 Daubert and Frye standards

   6.1.7.4 Legal aspects of ethanol and drugs of abuse

     6.1.7.4.1 Ethanol and drug-induced psychomotor impairment

     6.1.7.4.2 Collection/Storage/Interpretation of specimens

       6.1.7.4.3 Pharmacokinetics of ethanol and drugs of abuse

     6.1.7.4.4 Analysis

        6.1.7.4.4.1 Saliva

        6.1.7.4.4.2 Blood

        6.1.7.4.4.3 Breath

     6.1.7.4.5 Specific substances

        6.1.7.4.5.1 Ethanol

        6.1.7.4.5.2 Cannabis

### Part 7: ENVIRONMENTAL TOXICOLOGY

7.1 Background

7.1.1 Precautionary principle

7.1.2 Vulnerable populations

7.2 Chemical and Physical Exposures

7.2.1 Air pollutants

   7.2.1.1 Sources of air pollution

   7.2.1.2 Specific pollutants

      7.2.1.2.1 EPA criteria air pollutants (see 9.4.1 and 9.4.2)

      7.2.1.2.1.1 Carbon monoxide

      7.2.1.2.1.2 Oxides of nitrogen

      7.2.1.2.1.3 Ozone

      7.2.1.2.1.4 Particulate matter (PM2.5 & PM10)

      7.2.1.2.1.5 Sulfur dioxide

      7.2.1.2.1.6 Volatile organic compounds

     7.2.1.2.2 EPA hazardous air pollutants

     7.2.1.2.3 Indoor air pollutants

      7.2.1.2.3.1 Radon

      7.2.1.2.3.2 Tobacco smoke

      7.2.1.2.3.3 Volatile organic compounds

         7.2.1.2.3.3.1 Tight-building syndrome

      7.2.1.2.3.4 Mold

7.2.2 Water and soil pollutants

   7.2.2.1 Sources and sites of pollutants

   7.2.2.2 Specific water and soil pollutants

      7.2.2.2.1 Metals/Metalloids (see 9.5.2.9)

      7.2.2.2.2 Pesticides/Insecticides (see 9.5.2.10 and 9.5.2.11)

      7.2.2.2.3 Fungicides (see 9.5.2.10.2)

      7.2.2.2.4 Herbicides (see 9.5.2.10.3)

      7.2.2.2.5 Fertilizer (Nitrate/Nitrite)

      7.2.2.2.6 Perfluoroalkyl substances (PFAS)

      7.2.2.2.7 Persistent organic pollutants

        7.2.2.2.7.1 Dichlorodiphenyltrichloroethane (DDT)

        7.2.2.2.7.2 Polychlorinated biphenyls (PCB)

        7.2.2.2.7.3 Polychlorinated dibenzo-p-dioxins (PCDD/Dioxins)

        7.2.2.2.7.4 Polychlorinated dibenzofurans (PCDF)

      7.2.2.2.8 Petroleum-related

        7.2.2.2.8.1 Gasoline and additives

        7.2.2.2.8.2 Fracking

      7.2.2.2.9 Perchlorates

7.2.3 Food and drinking water toxicants

   7.2.3.1 Food safety

   7.2.3.2 Fish-related

    7.2.3.2.1 Bioaccumulation and biomagnification

    7.2.3.2.2 Specific pollutants

      7.2.3.2.2.1 Mercury (see 9.5.2.9.12)

      7.2.3.2.2.2 Polychlorinated biphenyls (PCBs) (see 7.2.2.2.7.2)

    7.2.3.3 Specific food/Drinking water additives and contaminants

      7.2.3.3.1 Metals/Metalloids (see 9.5.2.9)

      7.2.3.3.2 Antibiotics (see 2.1.2.1)

      7.2.3.3.3 Monosodium glutamate

      7.2.3.3.4 Endocrine-disrupting chemicals

        7.2.3.3.4.1 Bisphenols

        7.2.3.3.4.2 Phthalates (see 9.6.13.1.1)

      7.2.3.3.5 Pesticides (see 9.5.2.10)

      7.2.3.3.6 Fluoride

7.2.4 Symptoms attributed to the environment

   7.2.4.1 Multiple chemical sensitivity

   7.2.4.2 Gulf War Syndrome

   7.2.4.3 World Trade Center Health Program covered conditions

7.3 Natural Radiation Exposures

### Part 8: ADDICTION TOXICOLOGY AND SUBSTANCE USE

8.1 Definitions

8.1.1 Addiction

8.1.2 Dependence

8.1.3 Withdrawal

8.1.4 Tolerance

8.1.5 Hyperalgesia

8.2 Clinical Pharmacology of Drugs of Abuse (see 2.1.16)

8.2.1 Ethanol

8.2.2 Sedatives and GABA agents

8.2.3 Opioids/Opiates

8.2.4 Stimulants

8.2.5 Nicotine and tobacco

8.2.6 Cannabinoids

8.2.7 Hallucinogens

8.2.8 Dissociatives

8.2.9 Inhalants

8.3 Neurobiology of Addiction

8.4 Neuropharmacology of Addiction (see 1.2.5)

8.4.1 Acetylcholine

8.4.2 Norepinephrine

8.4.3 Dopamine

8.4.4 Serotonin

8.4.5 GABA

8.4.6 NMDA and glutamate receptors

8.4.7 Endogenous opioids

8.4.8 Endocannabinoids

8.5 Screening, Assessment, and Brief Intervention

8.6 Interpretation of Drug Testing (see 6.1.1 and 6.1.2.2)

8.7 Addiction Treatment

8.7.1 Alcohol use disorder

8.7.2 Substance use disorders

  8.7.2.1 Pharmacologic intervention for substance use disorders

     8.7.2.1.1 Opioid use disorder

        8.7.2.1.1.1 Buprenorphine

        8.7.2.1.1.2 Methadone

        8.7.2.1.1.3 Naltrexone

  8.7.2.2 Other substance abuse disorders

8.7.3 Behavioral interventions

8.8 Management of Intoxication From Substance Use (see 2.1.16)

8.9 Management of Withdrawal of Substance Use

8.9.1 Ethanol

8.9.2 Sedative-hypnotics

8.9.3 Nicotine

8.9.4 Opioids

8.9.5 Stimulants

8.9.6 Neonatal abstinence syndrome

### Part 9: OCCUPATIONAL TOXICOLOGY

9.1 Occupational Assessment and Prevention

9.1.1 Medical surveillance and pre-placement screening

9.1.2 Exposure control

  9.1.2.1 Engineering controls

  9.1.2.2 Personal protective equipment

  9.1.2.3 Work practices and product substitution

9.2 Occupational Monitoring

9.2.1 Biological monitoring and biomarkers

9.2.2 Environmental sampling/Exposure monitoring

9.3 Risk Assessment and Risk Management

9.3.1 Hazard identification

9.3.2 Dose response assessment

    9.3.2.1 No observed adverse effect levels (NOAEL)

     9.3.2.2 Lowest observed adverse effect levels (LOAEL)

    9.3.2.3 Benchmark dose

    9.3.2.4 Uncertainty factors

9.3.3 Exposure assessment

   9.3.3.1 Exposure pathways

   9.3.3.2 Interpretation of limit values

     9.3.3.2.1 Recommended Exposure Limit (REL)

     9.3.3.2.2 Permissible Exposure Limit (PEL)

     9.3.3.2.3 Threshold Limit Value (TLV)

9.3.4 Risk characterization

9.3.5 Risk perception and risk communication

9.4 Industrial Toxicants

9.4.1 Cellular asphyxiant gases (see 7.2.1.2.1)

   9.4.1.1 Carbon monoxide

   9.4.1.2 Cyanide

   9.4.1.3 Hydrogen sulfide

9.4.2 Irritant gases (see 7.2.1.2.1)

   9.4.2.1 Chlorine

   9.4.2.2 Nitrogen oxides

   9.4.2.3 Ozone

   9.4.2.4 Phosgene

   9.4.2.5 Sulfur oxides

9.4.3 Simple asphyxiants

9.5 Occupational Toxicants

9.5.1 Airborne solids

   9.5.1.1 Asbestos (see 9.6.11.2)

   9.5.1.2 Coal dust (see 9.6.11.1)

   9.5.1.3 Organic dust

   9.5.1.4 Silica (see 9.6.11.3)

   9.5.1.5 Synthetic mineral fibers

9.5.2 Hydrocarbons/Solvents/Fuels

   9.5.2.1 Aldehydes

     9.5.2.1.1 Acetaldehyde

     9.5.2.1.2 Formaldehyde

     9.5.2.1.3 Acrolein

   9.5.2.2 Alcohols and glycols

     9.5.2.2.1 Diethylene glycol

     9.5.2.2.2 Ethylene glycol

     9.5.2.2.3 Glycol ethers

     9.5.2.2.4 Isopropanol

     9.5.2.2.5 Methanol

   9.5.2.3 Aliphatic hydrocarbons (see 9.6.12.1)

     9.5.2.3.1 Hexane (see 9.6.19.1)

     9.5.2.3.2 Gasoline

     9.5.2.3.3 Kerosine

   9.5.2.4 Aromatic hydrocarbons (see 9.6.12.1)

     9.5.2.4.1 Benzene

     9.5.2.4.2 Polycyclic aromatic hydrocarbons

     9.5.2.4.3 Toluene

   9.5.2.5 Halogenated hydrocarbons

     9.5.2.5.1 Carbon tetrachloride

     9.5.2.5.2 Chloroform

       9.5.2.5.3 Methylene chloride

       9.5.2.5.4 Perchloroethylene

       9.5.2.5.5 Trichloroethylene

       9.5.2.5.6 Vinyl chloride (see 9.6.13.1.2 and 9.6.2.1.3)

    9.5.2.6 Hydrazines (see 9.6.8.2)

    9.5.2.7 Ketones

      9.5.2.7.1 Methyl ethyl ketone

      9.5.2.7.2 Diacetyl

      9.5.2.7.3 Acetone

    9.5.2.8 Peroxides (see 9.6.14.1)

      9.5.2.8.1 Hydrogen peroxide

      9.5.2.8.2 Sodium peroxide

    9.5.2.9 Metals/Metalloids (see 7.2.2.2.1, 9.6.1.3.2, 9.6.5.5, 9.6.9.1, and 9.6.18.2)

      9.5.2.9.1 Aluminum

      9.5.2.9.2 Antimony

      9.5.2.9.3 Arsenic

      9.5.2.9.4 Barium (see 9.5.2.12.5)

      9.5.2.9.5 Beryllium

      9.5.2.9.6 Cadmium

      9.5.2.9.7 Chromium

      9.5.2.9.8 Cobalt

      9.5.2.9.9 Copper

      9.5.2.9.10 Lead

      9.5.2.9.11 Manganese

      9.5.2.9.12 Mercury (see 7.2.3.2.2.1 and 9.6.10.4)

      9.5.2.9.13 Nickel

      9.5.2.9.14 Silver

      9.5.2.9.15 Thallium (see 9.5.2.12.10)

      9.5.2.9.16 Zinc

    9.5.2.10 Pesticides (see 7.2.2.2.2, 7.2.3.3.5, and 9.6.6.1)

      9.5.2.10.1 Fumigants (see 9.6.6.2)

      9.5.2.10.2 Fungicides (see 7.2.2.2.3)

      9.5.2.10.3 Herbicides (see 7.2.2.2.4)

    9.5.2.11 Insecticides and repellents (see 7.2.2.2.2)

      9.5.2.11.1 Carbamates

      9.5.2.11.2 Organochlorines

      9.5.2.11.3 Organophosphates

      9.5.2.11.4 Pyrethrins and pyrethroids

    9.5.2.12 Occupational rodenticides

      9.5.2.12.1 Coumarins and inandiones

      9.5.2.12.2 Sodium monofluoroacetate (SMFA)

      9.5.2.12.3 Zinc phosphide

      9.5.2.12.4 Arsenic trioxide

      9.5.2.12.5 Barium (see 9.5.2.9.4)

      9.5.2.12.6 Tetramine

      9.5.2.12.7 Cholecalciferol

      9.5.2.12.8 Bromethalin

      9.5.2.12.9 Alpha-Naphthylthiourea (ANTU)

      9.5.2.12.10 Thallium (see 9.5.2.9.15)

      9.5.2.12.11 Yellow phosphorus

      9.5.2.12.12 Strychnine

    9.5.2.13 Molluscicides

9.5.3 Nanotoxicology

9.6 Toxicants Associated with Specific Industry Exposures, Not Listed Elsewhere

9.6.1 Construction industry

   9.6.1.1 Carpentry and woodworking

   9.6.1.2 Paint/Painters

   9.6.1.3 Welding and metal joining

      9.6.1.3.1 Metal fume fever

      9.6.1.3.2 Metals/Metalloids (see 9.5.2.9)

   9.6.1.4 Roofing and road building

      9.6.1.4.1 Coal tar and pitch

9.6.2 Chemical industry

   9.6.2.1 Chemical manufacturing

      9.6.2.1.1 Acrylamide

      9.6.2.1.2 Acrylonitrile

      9.6.2.1.3 Vinyl chloride (see 9.6.13.1.2 and 9.5.2.5.6)

      9.6.2.1.4 Ethylene oxide (see 9.6.10.2)

      9.6.2.1.5 Polytetrafluoroethylene/Polymer fume fever

   9.6.2.2 Pharmaceutical manufacturing

      9.6.2.2.1 Dimethylformamide

      9.6.2.2.2 Ethylenediamine

      9.6.2.2.3 Isocyanates (see 9.4.2)

9.6.3 Cosmetology/Hair

   9.6.3.1 Paraphenylenediamine

   9.6.3.2 Perborates

9.6.4 Dry cleaning industry

   9.6.4.1 Trichloroethylene

   9.6.4.2 Perchloroethylene/Tetrachlorethylene

9.6.5 Explosives/Munitions/Fireworks manufacturing industry

   9.6.5.1 Dinitrotoluene

   9.6.5.2 Dinitrobenzene

   9.6.5.3 Chlorates

   9.6.5.4 Phosphorous

   9.6.5.5 Metals/Metalloids (see 9.5.2.9)

9.6.6 Agricultural/Farming industry

   9.6.6.1 Pesticides (see 9.5.2.10)

   9.6.6.2 Fumigants (see 9.5.2.10.1)

   9.6.6.3 Manure pits (see 9.4.1.3)

   9.6.6.4 Silos

9.6.7 Law enforcement/Military/Firefighting/EMS

   9.6.7.1 Combustion toxicology

   9.6.7.2 Riot control agents/Tear gas (see 2.8.2.6)

9.6.8 Aerospace industry

   9.6.8.1 Jet fuels

   9.6.8.2 Hydrazine rocket fuel (see 9.5.2.6)

9.6.9 Artists and artisans

   9.6.9.1 Metals/Metalloids (Lead/Cadmium/Manganese/Cobalt) (see 9.5.2.9)

   9.6.9.2 Solvents/Paint strippers

   9.6.9.3 Hydrofluoric acid (see 9.6.18.1)

9.6.10 Health care

   9.6.10.1 Acrylates

   9.6.10.2 Ethylene oxide (see 9.6.2.1.4)

   9.6.10.3 Nitrous oxide

   9.6.10.4 Mercury amalgam (see 9.5.2.9.12)

9.6.11 Mining industry

   9.6.11.1 Coal dust (see 9.5.1.2)

   9.6.11.2 Asbestos (see 9.5.1.1)

   9.6.11.3 Silica (see 9.5.1.4)

9.6.12 Petrochemical industry

   9.6.12.1 Aliphatic and aromatic hydrocarbons (see 9.5.2.3 and 9.5.2.4)

9.6.13 Plastics/Epoxy/Resins/Rubber manufacturing

   9.6.13.1 Plastics and foams

      9.6.13.1.1 Phthalates (see 7.2.3.3.4.2)

      9.6.13.1.2 Vinyl chloride (see 9.5.2.5.6 and 9.6.2.1.3)

      9.6.13.1.3 1,3-butadiene

      9.6.13.1.4 Dimethylformamide

      9.6.13.1.5 Organotins

   9.6.13.2 Epoxy and resins

      9.6.13.2.1 Epichlorohydrin

      9.6.13.2.2 Trimellitic anhydride

      9.6.13.2.3 Maleic anhydride

   9.6.13.3 Rubber manufacturing

      9.6.13.3.1 Ethylbenzene

      9.6.13.3.2 Butadiene

      9.6.13.3.3 Thiurams

      9.6.13.3.4 Dithiocarbamates

9.6.14 Pulp/Paper/Timber industry

   9.6.14.1 Peroxides (see 9.5.2.8)

   9.6.14.2 Mercaptans

   9.6.14.3 Wood dust

9.6.15 Sanitation and sewer treatment

   9.6.15.1 Hydrogen sulfide (see 9.4.1.3)

9.6.16 Semiconductor industry

   9.6.16.1 Fluorides

   9.6.16.2 Arsine/Stibine

   9.6.16.3 Phosphine

   9.6.16.4 Diborane

   9.6.16.5 Tetramethylammonium hydroxide

   9.6.16.6 Dichlorosilane/Trichlorosilane

9.6.17 Manufacture of ships and automobiles

   9.6.17.1 Styrene

   9.6.17.2 Sodium azide

9.6.18 Smelting/Metal refining/Reclamation

   9.6.18.1 Hydrofluoric acid (see 9.6.9.3)

   9.6.18.2 Metals/Metalloids (see 9.5.2.9)

9.6.19 Textile industry

   9.6.19.1 n-hexane (see 9.5.2.3.1)

   9.6.19.2 Cotton dust

   9.6.19.3 Carbon disulfide

   9.6.19.4 Dyes

      9.6.19.4.1 Aniline

      9.6.19.4.2 Benzidine

      9.6.19.4.3 Beta-naphthylamine

      9.6.19.4.4 Xylidine
